# The Political Vaudeville

**Published:** 1911-04

**Authors:** 


### EDITORIAL DEPARTMENT.

### THE POLITICAL VAUDEVILLE.

*Montes parturiunt nascitur ridiculus mus.*

The Thirty-second Legislature adjourned March 11th (ult.). The verdict is *"nihil fit,"*—unless I except that the vaudeville served to amuse the people of Austin, and added to the "gaiety of the nation." It was a veritable mine of fun for those sharp-witted young fellows sent here to report for the big dailies. The entire session of sixty days was taken up in the game of politics,—the "pros" and the "antis" sparring for wind, in the language of the ring; and, when the Bailey bluff was received, there was a panic amongst them, and they fell over each other in their eagerness to beg him to take it back. Despite Colquitt's lecture on riotous living and corn husks, despite his lash and his prayers "for the Lord's sake *do* something,"—they got away without making any appropriation for running the machine,—or redistricting the State, or relieving the Supreme Court or amending the penitentiary laws to correct the shameful mismanagement and the abuses under our infamous penal system,—or anything really useful. They would not stay long enough—after securing their *per diem,* to perfect any or many of the one thousand bills that had been set in motion; and—as to anything really useful, such as sanitary laws or laws in the interest of *race integrity,* which embrace a tremendous economic question; e. g., the bill prohibiting marriage of the unfit, and that providing for the arrest of hereditary insanity (published in our February number), they were hopelessly snowed under, as well as every bill seeking to regulate that monster evil—the saloon.

Well, two years more must elapse before a blow can be struck for health, virtue, morality and social economics, for—even if His Excellency should convene the immortal Thirty-second in extra session in August—it can consider none of these last named questions, but must confine itself to the appropriation and other bills especially submitted by him.

I have called it the "political vaudeville." The appropriateness of the name will be appreciated when it is explained that "vaudeville" means "the town calf" *(veau de ville)*—a Maypole festival in the Rhenish provinces of France to celebrate every spring the birth of the Burgomaster's calf,—he being the only person in the village able to own a cow, and she is the common property of the villagers. The "show" has surely been a source of amusement to the Austin people, and we would be in despair at its ending were we not consoled by the hope that it will soon be followed by Dr. Rucker's "Korak wonder"—the big tent show of all last summer—opening every night with a harangue by the "Doctor" on tapeworm. Hence, we dry our tears.

After the above was typed; after Colquitt had read the riot act to the squabblers, they got down to work (it took them ten days to cool down and fall in line), and really did perfect a few necessary things; for instance, the bill giving the provisional sanitary code prepared by the State Board of Health the force of law, so that it can be enforced by the Board; a compromise bill for a tuberculosis sanitarium (there were two bills), which appropriates $140,000 for two tent colonies for indigent consumptives, locations to be selected by a commission appointed by the Governor—and a superintendent for each, at a salary; and also a bill to prohibit drumming for doctors—under a penalty. These were all duly approved and signed by the Governor. Nothing was done to make effective the leprosy bill, passed by the Thirty-first Legislature; nor was the bill restricting the marriage of the unfit, nor the vasectomy bill ever taken up after the battle of the pigmies was over. They died a bornin', as did the absurd optometry bill introduced and championed by Dr. J. B. Hubbard, Representative from Kaufman, a member of the House Public Health Committee.

It is really touching—pathetic—to contemplate the solicitude of these tender-hearted lawmakers for the calves and lambs. They appropriated $100,000 to protect them from wolves. But the wolves in the guise of diphtheria, scarlet fever, pneumonia, fever, dysentery, may devour thousands of Texas babies, and not a dollar is forthcoming to enable the State Board to take their scalps.

What's the use of arguing? "Against stupidity—even the gods are powerless!"

